# Altered Directed Functional Connectivity of the Hippocampus in Mild Cognitive Impairment and Alzheimer’s Disease: A Resting-State fMRI Study

**DOI:** 10.3389/fnagi.2019.00326

**Published:** 2019-12-03

**Authors:** Jiayue Xue, Hao Guo, Yuan Gao, Xin Wang, Huifang Cui, Zeci Chen, Bin Wang, Jie Xiang

**Affiliations:** College of Information and Computer, Taiyuan University of Technology, Taiyuan, China

**Keywords:** hippocampus, Alzheimer’s disease, mild cognitive impairment, directed functional connectivity, resting-state fMRI

## Abstract

The hippocampus is generally reported as one of the regions most impacted by Alzheimer’s disease (AD) and is closely associated with memory function and orientation. Undirected functional connectivity (FC) alterations occur in patients with mild cognitive impairment (MCI) and AD, and these alterations have been the subject of many studies. However, abnormal patterns of directed FC remain poorly understood. In this study, to identify changes in directed FC between the hippocampus and other brain regions, Granger causality analysis (GCA) based on voxels was applied to resting-state functional magnetic resonance imaging (rs-fMRI) data from 29 AD, 65 MCI, and 30 normal control (NC) subjects. The results showed significant differences in the patterns of directed FC among the three groups. There were fewer brain regions showing changes in directed FC with the hippocampus in the MCI group than the NC group, and these regions were mainly located in the temporal lobe, frontal lobe, and cingulate cortex. However, regarding the abnormalities in directed FC in the AD group, the number of affected voxels was greater, the size of the clusters was larger, and the distribution was wider. Most of the abnormal connections were unidirectional and showed hemispheric asymmetry. In addition, we also investigated the correlations between the abnormal directional FCs and cognitive and clinical measurement scores in the three groups and found that some of them were significantly correlated. This study revealed abnormalities in the transmission and reception of information in the hippocampus of MCI and AD patients and offer insight into the neurophysiological mechanisms underlying MCI and AD.

## Introduction

Alzheimer’s disease (AD) is a common and progressive neurodegenerative disorder of the nervous system occurring in elderly individuals (Brookmeyer et al., 2007). AD is the most common form of dementia, which is characterized by memory impairment and cognitive function decline. Mild cognitive impairment (MCI) is generally considered an intermediate disease state between normal aging and AD and is characterized by significant cognitive impairment in the absence of dementia. MCI patients have a high risk of transitioning to AD. Previous studies have shown that approximately 10–15% of MCI patients per year progress to AD, whereas only 1–2% of cognitively normal aged people develop AD (Davatzikos, 2009). Dementia not only significantly affects the quality of life of elderly individuals but also entails heavy financial and psychological burdens for the family and society. Unlike AD, the irreversible pathophysiological changes in dementia can be delayed or prevented with early diagnosis and treatment. Therefore, it is crucial to develop accurate diagnostic methods and to study the pathological process underlying the transition from MCI to AD.

The hippocampus is generally considered one of the earliest affected brain regions in patients with AD (Braak and Braak, 1997). Various neuroimaging studies have demonstrated abnormal structure and function of the hippocampus in patients with AD and MCI. β-Amyloid abnormalities and tau pathology in the preclinical stages of AD initially appear in the hippocampus (Braak and Braak, 1991; Hanseeuw et al., 2016; Serra et al., 2018). Longitudinal studies have shown significant volume loss in the hippocampus during the progression of AD, and there is a direct relationship with cognitive decline (Everitt and Dunn, 2013; Joko et al., 2016). Altered functional connectivity (FC) of the hippocampus in patients with MCI and AD has attracted increasing attention from researchers. Wang et al. (2006) studied connectivity changes between the hippocampus and all other brain regions in AD patients and found disrupted FC between the hippocampus and a number of regions including the medial prefrontal cortex, ventral anterior cingulate cortex, right inferotemporal cortex, right superior and middle temporal gyrus, and posterior cingulate cortex. Kim et al. (2013) used structural and fMRI data to examine the aberrant patterns of FC between the hippocampus and the rest of the brain and discovered reduced FC between the hippocampus and precuneus in AD patients. Sohn et al. (2014) investigated FC between the hippocampus and several specific brain regions in amnestic MCI and AD patients using rs-fMRI data. Tahmasian et al. (2015) performed seed-based FC analyses of the hippocampus in subjects with MCI or AD and normal controls (NCs). The results indicated that the intrinsic connectivity between the hippocampus and precuneus was significantly reduced, and hippocampal glucose metabolism was increased in the patient groups. Sun et al. (2017) studied FC of the medial temporal brain regions in MCI, AD, and NC subjects and found a significant difference in FC between the bilateral perirhinal cortex and right hippocampus.

Functional connectivity only reflects the interactions between different brain regions, but effective connectivity is able to explore the direction and intensity of information flow in the interacting brain regions and offers a better understanding of the interaction patterns of different brain regions. Liu et al. (2012) investigated effective connectivity between resting state networks in AD patients and NCs and found that the interactions between resting-state networks in AD patients and NCs were different. Zhong et al. (2014) found differences in effective connectivity of the default mode network (DMN) between AD patients and NCs. The most common methods for studying effective connectivity include structural equation models (SEMs), dynamic causal modeling (DCM), and Granger causality analysis (GCA). The SEMs and DCM are based on theoretical assumptions and specify hypotheses in terms of different models or architectures (Bajaj et al., 2016). Unlike SEMs and DCM, GCA does not require theoretical assumptions about the existence and direction of influence between any two regions. The need for theoretical assumptions also means that SEMs and DCM may lead to erroneous conclusions due to any misspecification. Additionally, the models required for SEMs and DCM cannot represent networks with large numbers of nodes (Roebroeck et al., 2005; Zhao et al., 2016; Zhang et al., 2017), and GCA is sufficiently flexible to accommodate hemodynamic variability (Bressler and Seth, 2011).

Numerous studies have demonstrated the effectiveness of measuring the directed connectivity of fMRI time series with GCA (Liao et al., 2011; Wen et al., 2013; Liang et al., 2014; Khazaee et al., 2017). Liang et al. (2014) identified the patterns of effective connectivity among four important resting-state networks, i.e., the DMN, hippocampal–cortical memory network, frontoparietal control network, and dorsal attention network, via GCA in amnestic MCI patients. Those researchers found that the directed connections between areas in the four networks were significantly altered in aMCI patients compared with healthy controls. Khazaee et al. (2017) applied GCA to construct the whole-brain directed network with rs-fMRI data from MCI, AD, and NC subjects and subsequently performed a classification of the three groups using directed graph measures. An accuracy of 93.3% was eventually achieved. A large number of studies have shown that FC between the hippocampus and other brain regions was abnormal in MCI and AD patients, and these abnormalities might be associated with the pathology of MCI and AD. However, little research has examined the abnormal directed connectivity of the hippocampus in MCI and AD patients, and therefore, the alterations in the directed connectivity patterns of the hippocampus in MCI and AD patients are largely unknown.

In the current study, to identify the directed connectivity changes in the brain related to AD progression, we applied GCA to explore the resting-state-directed connectivity of the hippocampus in MCI and AD patients. We used the hippocampus as the seed region and detected directed FC between the hippocampus and other brain regions using voxel wise GCA.

## Materials and Methods

### Participants

All of the data used in this study were obtained from the Alzheimer’s Disease Neuroimaging Initiative (ADNI) database^1^. The purpose of the ADNI is to study the pathogenesis and prevention of AD by analyzing a variety of types of medical imaging data. The rs-fMRI data of 29 patients with AD (average age = 72.33 years; 18 females), 65 patients with MCI (average age = 71.8 years; 29 females), and 30 NC subjects (average age = 74.18 years; 19 females) (Table 1) were analyzed in this study. All participants were right-handed and had no history of neurological or psychiatric disorders. The patients with AD had a Mini-Mental State Examination (MMSE) score of 14–26, a Clinical Dementia Rating (CDR) of 0.5 or 1.0 and met the National Institute of Neurological and Communicative Disorders and Stroke and the Alzheimer’s Disease and Related Disorders Association (NINCDS/ADRDA) criteria for probable AD. The MCI patients had MMSE scores of 23–30 and CDR scores of 0.5. The NC subjects were non-depressed, non-MCI, and non-demented and had MMSE scores of 24–30 and CDR scores of 0. To assess test–retest reliability, 19 patients with AD, 37 patients with MCI, and 19 NC subjects were scanned twice. The mean interval between two scans was 3 months.

**TABLE 1:** Demographic and clinical data.

	**NC**	**MCI**	**AD**	***p*-value**
Number	30	65	29	
Sex (M/F)	11/19	36/29	11/18	0.137^a^
Age (years)	74.18 ± 5.96	72.29 ± 7.1	72.33 ± 7.26	0.438^*b*^
MMSE	28.91 ± 1.7	27.29 ± 2.39	21 ± 3.52	<0.001^b^
CDR	0.07 ± 0.17	0.51 ± 0.15	0.89 ± 0.21	<0.001^b^
FAQ	0.22 ± 0.83	4.08 ± 4.76	18 ± 6.4	<0.001^b^

*Data represent the mean ± SD. MMSE, Mini-Mental State Examination; CDR, Clinical Dementia Rating; FAQ, functional activities questionnaire. ^*a*^The *p*-value was obtained using a two-tailed Pearson chi-square test. ^*b*^The *p*-value was obtained by one-way analysis of variance.*

### Data Acquisition and Preprocessing

All subjects underwent rs-fMRI in a 3T MR scanner (Siemens Trio 3-Tesla scanner, Siemens, Erlangen, Germany). During the scan, participants were asked to relax with their eyes closed but not to fall asleep (Jack et al., 2008). Each scan consisted of 140 contiguous EPI functional volumes with the scan parameters set as follows: slice number = 48, slice thickness = 3.3 mm, repetition time (TR) = 3,000 ms, echo time (TE) = 30 ms, field of view = 192 × 192 mm, matrix = 64 × 64 mm, and flip angle = 80°. Preprocessing of the rs-fMRI data was performed using the Data Processing Assistant for Resting-State fMRI (DPARSF) toolbox (Yan and Zang, 2010) and the SPM8 package^2^. The preprocessing steps were applied as follows: the first 10 volumes of the time series data were discarded due to signal equilibrium and allow the participants to adapt to the scanning noise; slice timing was applied to compensate for the time difference between different slices in each volume; realignment for head movement compensation was applied using a six-parameter rigid-body spatial transformation; all images were normalized into the Montreal Neurological Institute (MNI) space; the resulting images were detrended to reduce the effects of low-frequency drift, smoothed using a Gaussian filter with FWHM = 4 mm, and bandpass filtered (0.01–0.08 Hz) to remove high frequency physiological noise; and finally, a Friston-24 parameter and parameters for the global mean signal, cerebrospinal fluid signal, and white matter signal were regressed out as nuisance covariates for the subsequent analysis (Kelly et al., 2008).

### Definition of Seed Regions

**FIGURE 1:**
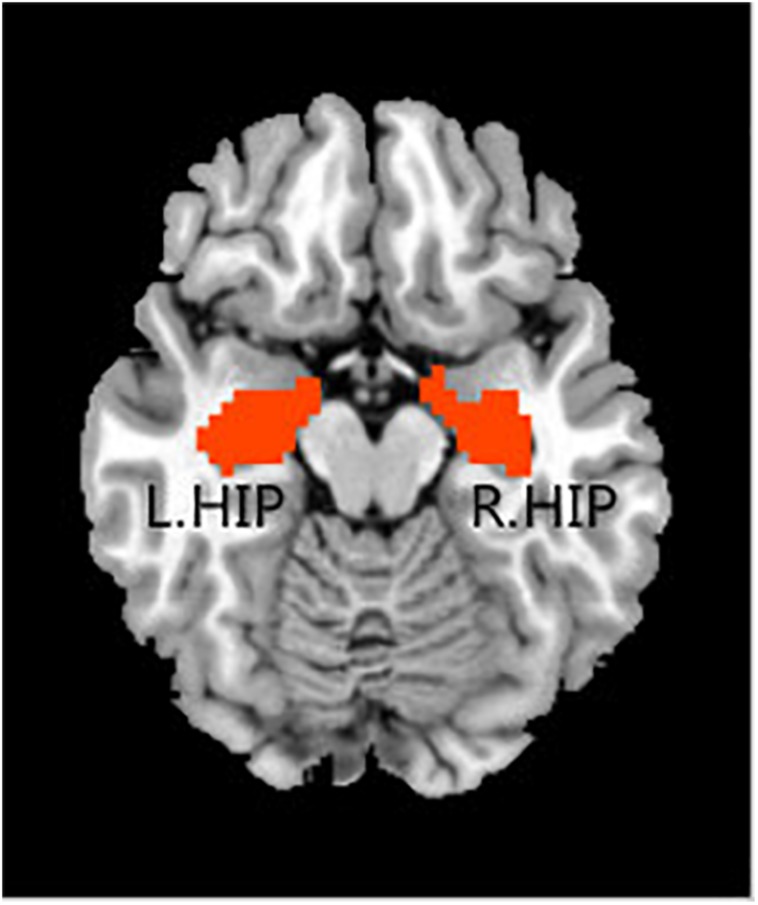
Regions of interest, including the left hippocampus and right hippocampus.

In the rs-fMRI data analysis for each individual, the bilateral hippocampal ROIs were defined using free software in the rs-fMRI data analysis toolkit REST^3^. The ROIs were extracted from the Anatomical Automatic Labeling (AAL) template (Tzourio-Mazoyer et al., 2002). For each seed region, the BOLD time series of the voxels within the seed region were averaged to generate the reference time series for this seed region (Figure 1).

### Directed Brain Functional Connectivity

Multivariate GCA was applied to each voxel in the whole brain to obtain the directed brain FC from the seed points to other voxels in the whole brain and the reverse impact intensities from other voxels in the whole brain to the seed points. The signed-path multivariate Granger causality coefficients of each pair of time series were calculated using REST-GCA software (Zang et al., 2012). The bivariate Granger causality of two time series *X*(*t*) and *Y*(*t*) can be formulated by a vector autoregressive model (Hamilton et al., 2009):

(1) $$Y_{t}=\sum_{i=1}^{p}A_{i}\,X_{\left(t-i\right)}+\sum_{i=1}^{p}B_{i}\,Y_{\left(t-i\right)}+C\,Z_{t}+\varepsilon_{t}$$

(2) $$X_{t}=\sum_{i=1}^{p}A_{i}^{\prime }\,Y_{\left(t-i\right)}+\sum_{i=1}^{p}B_{i}^{\prime }\,X_{t-i}+C^{\prime }\,Z_{t}+\varepsilon_{t}^{\prime }$$

where *p* is the model order, i.e., the maximum number of lagged samples, *A′_*i*_* [formula (1)] and *A*_*i*_ [formula (2)] are the signed-path coefficients, *B*_*i*_ [formula (1)] and *B′_*i*_* [formula (2)] are autoregression coefficients, ε*_*t*_* [formula (1)] and ε′*_*t*_* [formula (2)] are residuals, and *Z*_*t*_ represents the covariates (e.g., head motion, global trend, and time series from certain brain areas). The time series *X*_*t*_ significantly Granger-causes the time series *Y*_*t*_ if the signed-path coefficient *A*_*i*_ is significantly larger or smaller than zero, and vice versa, i.e., *Y*_*t*_ can be defined as a significant Granger-cause to *X*_*t*_ if the signed-path coefficient *A′_*i*_* is significantly larger or smaller than zero. Multivariate Granger causality is a generalization of the above bivariate model by including more than two variables.

### Statistical Analysis

Demographic and clinical data were analyzed using the Statistical Package for Social Sciences (SPSS 20.0) software. Continuous variables are presented as the means and standard deviations (SD). Categorical data are presented as numbers and percentages. Group differences in sex were assessed with a two-tailed Pearson chi-square test, and the remaining variables were tested using one-way analyses of variance (ANOVA) with group (NC, MCI, AD) as the between-subjects factor. All *p*-values of <0.05 were considered statistically significant.

The GCA FC maps were analyzed based on voxel wise comparisons across the whole brain, which was accomplished using the Statistical Analysis function in the REST software. The calculated intensity values were normalized (Fisher *z*-transformation), and two-sample *t*-tests were applied to identify regions showing significant differences in directed FC between any two groups with age and sex removed as covariates. The threshold for significant differences between groups was set to *p* < 0.05 with a minimum cluster size of 85 contiguous voxels (Zheng et al., 2017).

Spearman’s correlation was used to assess the relationship between the GCA coefficients for the abnormal directional connections and MMSE, FAQ, and CDR in the three groups using SPSS 20.0 software.

### Test–Retest Reliability

Consistent with previous research (Braun et al., 2012; Plichta et al., 2012; Cao et al., 2014), we calculated a commonly used index, namely, the intraclass correlation coefficient (ICC) (Shrout and Fleiss, 2015), to investigate the test–retest reliability of the abnormal directional connections between the three groups. The ICC values were calculated according to the following equation:

(3) $$I\,C\,C=\frac{M\,S_{\lambda }-M\,S_{\varepsilon }}{M\,S_{\lambda }+\left(k-1\right)\,M\,S_{\varepsilon }+\frac{k}{n}\,\left(M\,S_{\pi }-M\,S_{\varepsilon }\right)}$$

where *MS*_λ_ is the between-subject mean square, *MS*_ε_ is the residual mean square, *MS*_π_ is the between-scan mean square, *n* is the number of subjects, and *k* is the number of repeated observations per subject. Here, ICC values close to 1 indicate perfect agreement between the two scans, while ICC values close to 0 (or negative) indicate poor or no agreement. In this study, we used a standard scale to classify reliability (Winer, 1962; Sampat et al., 2006), with an ICC value from 0 to 0.25 indicating poor reliability; 0.25 to 0.4 indicating low reliability; 0.4 to 0.6 indicating fair reliability; 0.6 to 0.75 indicating good reliability; and 0.75 to 1.0 indicating excellent reliability.

## Results

### Demographic and Clinical Data

The demographic and clinical data for each group are presented in Table 1. No significant differences were observed in sex or age among the three groups (*p* > 0.05). However, the MMSE, CDR, and FAQ scores were significantly different (*p* < 0.001, Bonferroni correction) among the three groups.

### Altered Hippocampal Directed Functional Connectivity: Pairwise Comparisons

**FIGURE 2:**
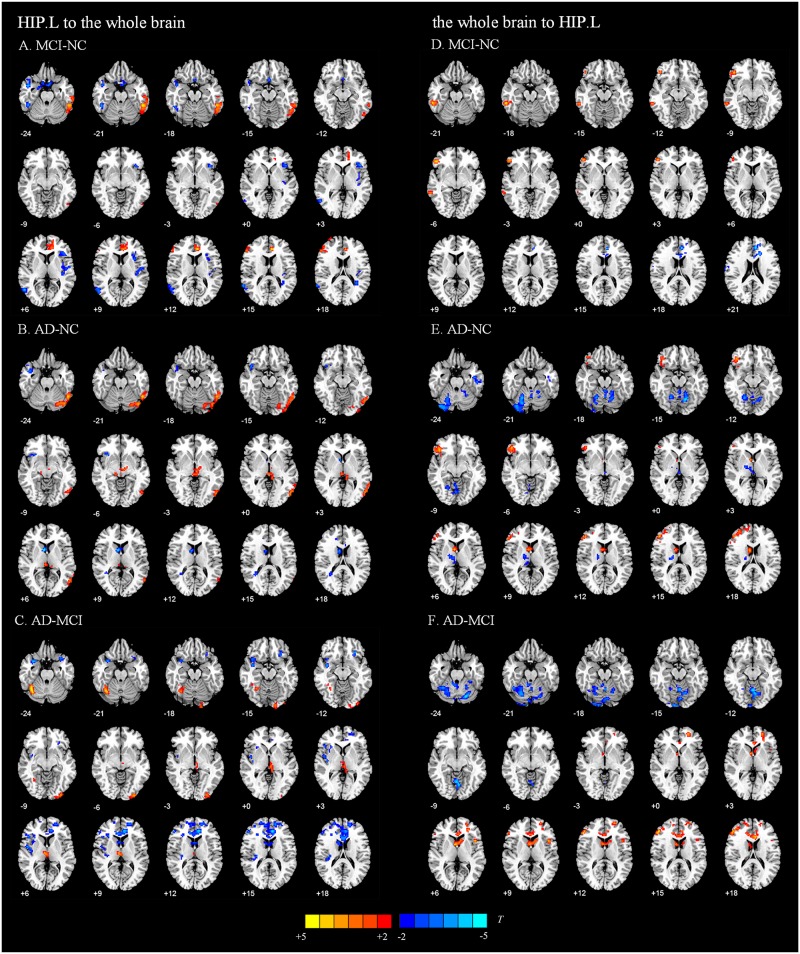
Directed functional connectivity showing significant differences in the left hippocampus and whole brain pairwise comparisons. From the left hippocampus to whole brain, panel **(A)** represents the directed functional connectivity showing significant differences between the MCI and NC groups, panel **(B)** represents the directed functional connectivity showing significant differences between the AD and NC groups, and panel **(C)** represents the directed functional connectivity showing significant differences between the AD and MCI groups. From whole brain to the left hippocampus, panel **(D)** represents the directed functional connectivity showing significant differences between the MCI and NC groups, panel **(E)** represents the directed functional connectivity showing significant differences between the AD and NC groups, and panel **(F)** represents the directed functional connectivity showing significant differences between the AD and MCI groups. The red/yellow-colored brain regions indicate increased GCA values between groups. The blue/green-colored brain regions indicate decreased GCA values between groups.

**FIGURE 3:**
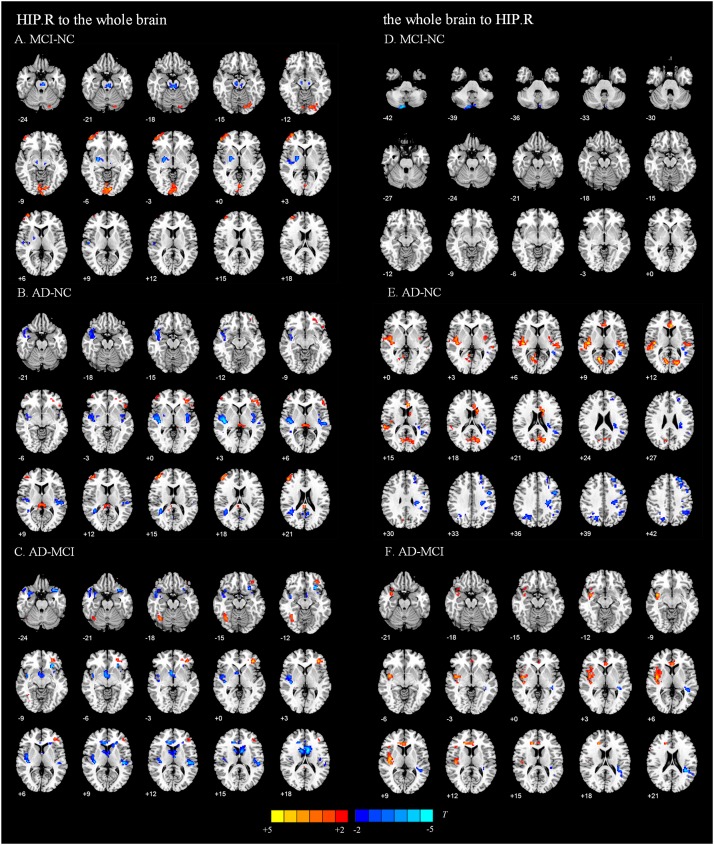
Directed functional connectivity showing significant differences in the right hippocampus and whole brain pairwise comparisons. From the right hippocampus to whole brain, panel **(A)** represents the directed functional connectivity showing significant differences between the MCI and NC groups, panel **(B)** represents the directed functional connectivity showing significant differences between the AD and NC groups, and panel **(C)** represents the directed functional connectivity showing significant differences between the AD and MCI groups. From whole brain to the right hippocampus, panel **(D)** represents the directed functional connectivity showing significant differences between the MCI and NC groups, panel **(E)** represents the directed functional connectivity showing significant differences between the AD and NC groups, and panel **(F)** represents the directed functional connectivity showing significant differences between the AD and MCI groups. The red/yellow-colored brain regions indicate increased GCA values between groups. The blue/green-colored brain regions indicate decreased GCA values between groups.

We present the average GCA coefficients for the abnormal directional connections between the hippocampus and the whole brain within each group (NC, MCI, and AD). The results are shown in Supplementary Tables S1, S2. The pairwise comparisons of differences in hippocampal connectivity were obtained using the REST two-sample *t*-tests. In the directed FC from the left hippocampus to the whole brain, the MCI group, compared with the NC group, showed significantly decreased connectivity to the right inferior temporal gyrus, right middle temporal gyrus, right parahippocampal gyrus, and part of the prefrontal cortex. Enhanced connectivity was detected from the left hippocampus to the left inferior temporal gyrus, left anterior cingulate and paracingulate gyri, and right inferior frontal gyrus, triangular portion (Figure 2A and Table 2). Compared with the NC group, the AD group showed strengthened connectivity from the left hippocampus to the left inferior temporal gyrus and left precuneus and weakened connectivity from the left hippocampus to the right temporal pole: superior temporal gyrus, right caudate nucleus, right posterior cingulate gyrus, and right superior frontal gyrus (Figure 2B and Table 2). Compared with the MCI group, the AD group demonstrated increased connectivity from the left hippocampus to regions of the right cerebellum, left middle occipital gyrus, and right thalamus and decreased connectivity from the left hippocampus to the bilateral temporal pole, superior temporal gyrus, right rolandic operculum, left anterior cingulate and paracingulate gyri, and left superior frontal gyrus (Figure 2C and Table 2). The differences between groups in directed FC from the whole brain to the left hippocampus are shown in Figures 2D–F and Table 2. The detailed results of the pairwise comparisons regarding the directed FC between the right hippocampus and the whole brain are displayed in Figure 3 and Table 3.

**TABLE 2:** Directed functional connectivity showing significant differences in the left hippocampus and the whole brain pairwise comparisons.

**Brain region**	**Abbreviation**	**Peak coordinate (MNI)**			***T*-score**	**Cluster size (voxels)**
		***X***	***Y***	***Z***		
**Left HIP to the whole brain**						
NC vs. MCI						
Right inferior temporal gyrus	ITG.R	48	−45	−24	−3.3837	109
Left inferior temporal gyrus	ITG.L	−54	−45	−21	4.4304	245
Right middle temporal gyrus	MTG.R	45	3	−27	−4.2429	117
Right parahippocampal gyrus	PHG.R	21	−3	−27	−3.0788	122
Left inferior frontal gyrus, triangular portion	IFGtriang.L	−42	24	3	−3.17	98
Left anterior cingulate and paracingulate gyri	ACG.L	−6	36	15	4.2995	119
Right inferior frontal gyrus, triangular portion	IFGtriang.R	54	33	15	3.0303	144
Left middle frontal gyrus	MFG.L	−45	12	51	−3.5591	91
Right superior frontal gyrus	SFG.R	18	6	63	−3.8702	90
NC vs. AD						
Right temporal pole: superior temporal gyrus	TPOsup.R	42	3	−27	−3.9766	173
Left inferior temporal gyrus	ITG.L	−54	−57	−24	4.0115	481
Right caudate nucleus	CAU.R	6	9	6	−3.6686	121
Right posterior cingulate gyrus	PCG.R	12	−39	27	−3.1048	162
Right superior frontal gyrus	SFG.R	15	36	33	−3.8617	388
Left precuneus	PCUN.L	−9	−63	63	4.443	259
MCI vs. AD						
Left temporal pole: superior temporal gyrus	TPOsup.L	−27	3	−33	−3.2968	121
Right insula	INS.R	39	0	−12	−3.6394	115
Right cerebellum_6	Cereb.R	39	−57	−24	4.079	161
Left middle occipital gyrus	MOG.L	−30	−96	−6	3.077	91
Right thalamus	THA.R	6	−15	6	3.1359	87
Right rolandic operculum	ROL.R	48	0	6	−3.1668	97
Left anterior cingulate and paracingulate gyri	ACG.L	−6	18	24	−4.2899	1419
Left superior frontal gyrus	SFG.L	−15	54	15	−3.2588	180
**Whole brain to the left HIP**						
NC vs. MCI						
Right inferior temporal gyrus	ITG.R	60	−42	−18	3.251	108
Right inferior frontal gyrus, orbital portion	ORBinf.R	45	36	−12	3.5497	93
Left superior frontal gyrus, medial	SFGmed.L	−6	42	21	−3.6127	203
Right supplementary motor area	SMA.R	9	0	63	3.424	139
NC vs. AD						
Left cerebellum_9	Cereb.L	−9	−57	−57	−3.513	163
Left cerebellum_Crus2	Cereb.L	−48	−69	−39	−3.2864	91
Right cerebellum_6	Cereb.R	21	−60	−21	−3.6537	253
Left cerebellum_6	Cereb.L	−15	−66	−15	−3.9553	200
Left inferior temporal gyrus	ITG.L	−60	−12	−33	−4.1771	114
Right inferior frontal gyrus, triangular portion	IFGtriang.R	48	30	27	4.6348	914
Right caudate nucleus	CAU.R	6	6	6	4.0654	99
Right superior frontal gyrus	SFG.R	24	9	57	3.5079	185
MCI vs. AD						
Left cerebellum_Crus2	Cerebe_Crus2.L	−27	−78	−57	−3.6233	98
Left cerebellum_Crus1	Cerebe_Crus1.L	−21	−75	−24	−3.8854	684
Left anterior cingulate and paracingulate gyri	ACG.L	−6	24	18	3.6713	263
Left superior frontal gyrus	SFG.L	−24	51	0	3.2931	91
Right caudate nucleus	CAU.R	3	6	6	3.6922	195
Left inferior frontal gyrus, triangular portion	IFGtriang.L	−45	15	6	3.6202	158
Right inferior frontal gyrus, triangular portion	IFGtriang.R	51	36	15	4.3022	672

**TABLE 3:** Directed functional connectivity showing significant differences in the right hippocampus and the whole brain pairwise comparisons.

**Brain region**	**Abbreviation**	**Peak coordinate (MNI)**			***T*-score**	**Cluster size (voxels)**
		***X***	***Y***	***Z***		
**Right HIP to the whole brain**						
NC vs. MCI						
Right lenticular nucleus, pallidum	PAL.R	−6	−21	−24	−3.5274	237
Left calcarine fissure and surrounding cortex	CAL.L	−9	−90	−6	3.2123	223
Right middle frontal gyrus	MFG.R	36	60	3	3.0021	154
Left paracentral lobule	PCL.L	−6	−33	51	−3.4208	113
NC vs. AD						
Left Vermis_10	Vermis_10. L	−3	−42	−36	−3.4724	134
Right insula	INS.R	39	−18	3	−5.1014	433
Left inferior frontal gyrus, triangular portion	IFGtriang.L	−36	36	0	3.1908	92
Left superior temporal gyrus	STG.L	−54	−24	6	−3.6128	153
Right middle frontal gyrus	MFG.R	39	51	15	3.708	116
Right median cingulate and paracingulate gyri	DCG.R	3	−24	30	3.7886	195
Left precentral gyrus	PreCG.L	−51	0	33	4.3147	91
Left inferior parietal, but supramarginal and angular gyri	IPL.L	−30	−75	48	3.1726	99
Right precuneus	PCUN.R	12	−63	63	3.6249	137
MCI vs. AD						
Right Heschl gyrus	HES.R	39	−21	6	−3.4666	327
Right fusiform gyrus	FFG.R	36	−63	−15	3.3393	112
Left insula	INS.L	−27	21	−12	−4.0594	106
Left inferior frontal gyrus, triangular portion	IFGtriang.L	−36	36	0	3.5881	140
Right caudate nucleus	CAU.R	6	6	−3	−3.4331	94
Left caudate nucleus	CAU.L	−9	9	18	−3.8284	464
Left superior temporal gyrus	STG.L	−45	−27	9	−3.8611	107
Right median cingulate and paracingulate gyri	DCG.R	24	−42	42	4.0994	379
Right precuneus	PCUN.R	15	−78	48	4.1331	696
**Whole brain to the right HIP**						
NC vs. MCI						
Right cerebellum_Crus2	Cerebe_Crus2.R	9	−87	−42	−3.6594	103
NC vs. AD						
Right cerebellum_Crus2	Cerebe_Crus2.R	18	−78	−57	−3.0899	107
Right Heschl gyrus	HES.R	42	−21	9	3.9572	313
Left middle temporal gyrus	MTG.L	−45	−48	18	−3.4141	173
Right calcarine fissure and surrounding cortex	CAL.R	12	−63	9	3.874	284
Left anterior cingulate and paracingulate gyri	ACG.L	−6	27	15	3.949	107
Left middle frontal gyrus	MFG.L	−30	24	51	−4.9485	434
Right superior occipital gyrus	SOG.R	24	−69	39	−3.2264	93
MCI vs. AD						
Right insula	INS.R	39	0	−9	3.8809	394
Left anterior cingulate and paracingulate gyri	ACG.L	−18	33	18	3.4111	107
Left superior temporal gyrus	STG.L	−36	−42	21	−3.8151	148
Right inferior frontal gyrus, opercular portion	IFGoperc.R	60	15	27	3.1223	89
Right precuneus	PCUN.R	18	−75	45	−3.2829	173
Left middle frontal gyrus	MFG.L	−33	24	51	−3.5317	86
Right paracentral lobule	PCL.R	6	−21	78	−3.8933	142

**FIGURE 4:**
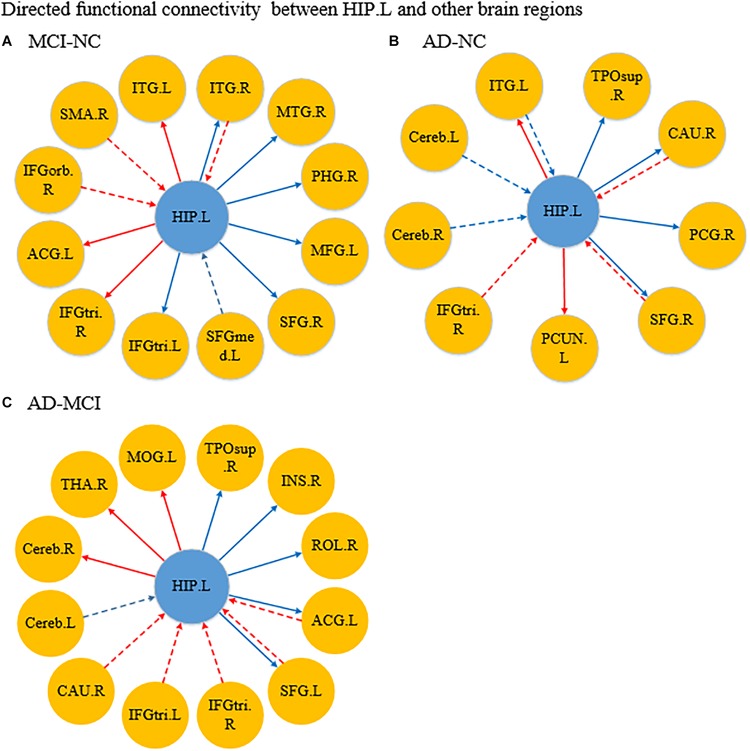
Differences in directed functional connectivity from the left hippocampus to the whole brain and from the whole brain to the left hippocampus between each pair of groups. Panel **(A)** represents the comparison between the MCI and NC groups, panel **(B)** represents the comparison between the AD and NC groups, and panel **(C)** represents the comparison between the AD and MCI groups. The red line denotes that the GCA value in the former group was higher than the latter group, and the blue line denotes that the GCA value in the former group was lower than the latter group. The solid line indicates the directed functional connectivity from the hippocampus to other brain regions, and the dashed line indicates the directed functional connectivity from other brain regions to the hippocampus.

**FIGURE 5:**
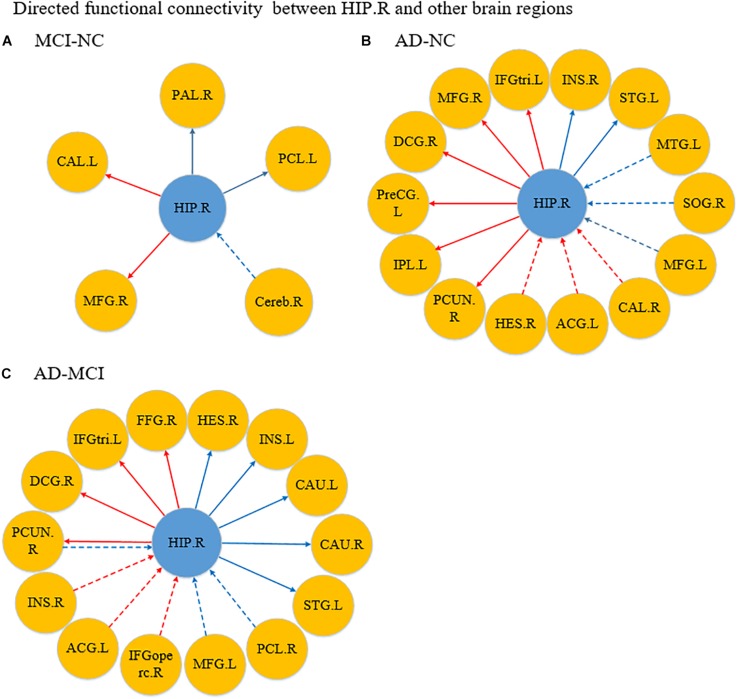
Differences in directed functional connectivity from the right hippocampus to the whole brain and from the whole brain to the right hippocampus between each pair of groups. Panel **(A)** represents the comparison between the MCI and NC groups, panel **(B)** represents the comparison between the AD and NC groups, and panel **(C)** represents the comparison between the AD and MCI groups. The red line denotes that the GCA value in the former group was higher than the latter group, and the blue line denotes that the GCA value in the former group was lower than the latter group. The solid line indicates the directed functional connectivity from the hippocampus to other brain regions, and the dashed line indicates the directed functional connectivity from other brain regions to the hippocampus.

Figure 4A shows the significantly altered directed FC between the left hippocampus and the whole brain in the MCI group compared with the NC group. The difference focuses primarily on the connectivity from the left hippocampus to other brain regions. The connectivity from the left hippocampus to the right middle temporal gyrus and the right cerebellum was decreased, whereas the left inferior temporal gyrus, left anterior cingulate and paracingulate gyri, and the middle frontal gyrus all showed enhanced directed FC to the left hippocampus to varying degrees. Figure 4B indicates the difference in the directed FC between the left hippocampus and other brain regions in the NC group and the AD group. The connectivity from the left hippocampus to the left inferior temporal gyrus was demonstrated to be enhanced in the AD group, which was similar to the findings in the MCI group. However, the differences mainly occurred in the connectivity from other brain regions to the left hippocampus. Figure 4C shows the differences in directed FC between the left hippocampus and the whole brain in the MCI and AD groups. A large number of abnormal connections were identified, but this analysis mostly showed decreased directed connectivity. Figure 5A illustrates the significantly altered directed FC between the right hippocampus and the whole brain in the MCI group compared with the NC group. There were few abnormal connections. Figure 5B indicates the difference in the directed FC between the right hippocampus and other brain regions in the NC group and the AD group. The differences are primarily found in the connectivity from the right hippocampus to other brain regions. Figure 5C shows the differences in the directed FC between the right hippocampus and the whole brain in the MCI and AD groups. A large number of abnormal connections were noted, but these were mainly concentrated in regions that received information from the right hippocampus.

### Relationships Between the Abnormal Directional Connections and Cognitive and Clinical Measurements

Simultaneously, in order to explore the potential relationship between the abnormal directional connections and cognitive and clinical measurements, we found some significant correlations between the GCA coefficients for the abnormal directional connections and MMSE, CDR, and FAQ in the three groups. The results are shown in Supplementary Tables S3, S4. For example, the GCA coefficients from the left hippocampus to left inferior temporal gyrus (*r* = −0.214, *p* = 0.045) and to left precuneus (*r* = −0.300, *p* = 0.025) were negatively correlated with MMSE in the three groups, and the GCA coefficient from the left hippocampus to left anterior cingulate and paracingulate gyri was positively correlated with CDR (*r* = 0.286, *p* = 0.006) and FAQ (*r* = 0.301, *p* = 0.004) in NC and MCI groups. Moreover, the GCA coefficients from the right hippocampus to right middle frontal gyrus, from right superior frontal gyrus to the left hippocampus, and from left middle frontal gyrus to the right hippocampus were significantly correlated with MMSE, CDR, and FAQ in NC and AD groups (showed in Supplementary Tables S3, S4).

### Test–Retest Reliability of the Abnormal Directional Connections Between the Three Groups

To further understand the reliability of the GCA differences among the three groups, we calculated the ICC values of the abnormal directional connections among the three groups and found fair average reliability of the abnormal directional connections (showed in Supplementary Figures S1, S2). For instance, our study found that the interconnection between the left hippocampus and inferior temporal gyrus showed good reliability in NC and MCI groups. Moreover, the directional FC from the left hippocampus to right middle temporal gyrus, left anterior cingulate and paracingulate gyri, left middle frontal gyrus, right superior frontal gyrus, and right inferior frontal gyrus, triangular portion showed low to good reliability in NC and MCI groups.

## Discussion

In the current study, we used the hippocampus (HIP) as connective node and applied the GCA coefficient to investigate the directed relationship between the HIP and the whole brain. The results revealed alterations in the directed FCs from the HIP to the whole brain and from the whole brain to the HIP in patients with MCI and AD. Furthermore, significant differences among the three groups were mainly found in the temporal lobe, frontal lobe, and cingulate cortex. Within these regions, the main brain regions showing abnormalities included ITG, right PCUN, MTG, left STG, MFG, IFG, left ACG, and right INS. Notably, most of these differences were significantly correlated with cognitive and clinical measurements in the three groups.

### Abnormal Directed Connectivity Between the HIP and the Default Mode Network

Most of the above regions belong to the DMN, which is considered responsible for episodic memory formation and attention (Cechetto and Weishaupt, 2016). The DMN is thought to be primarily responsible for internally focused thought processes, such as autobiographical memory and experience of the self. Previous studies have consistently demonstrated that the DMN is the first network to be affected in AD, and decreased DMN FC is known to occur in AD patients (Ashwal, 2017; Brown et al., 2019).

Specifically, our study found the directed FCs from the left HIP to left ITG and from the right HIP to right PCUN in MCI and AD patients were stronger than NCs. Actually, the ITG is located at the end of the ventral visual stream (Tanaka, 1996) and is considered relevant to long-term visual memory storage (Miyashita, 1993) and cognitive learning. Furthermore, the PCUN is also critical to visuospatial imaging, retrieval of episodic memory and information processing of self-related information (Cavanna and Trimble, 2006; Hebscher et al., 2017; Mazzoni et al., 2019). Hence, this finding may indicate that these abnormal FCs might be associated with cognitive decline and visuospatial disability in MCI and AD patients. Notably, according to the research by Convit et al. (2000), the ITG might be the first temporal lobe neocortical site affected in AD, and atrophy in this region could be used to distinguish AD from non-demented individuals. This may be related to the fact that the directed FC from left ITG to the left HIP only weakens in AD patients, and may mean that ITG have not fully atrophy during the MCI group compared to the AD group.

Comparing with NCs, the directed FC between the HIP and MTG was significantly decreased in patients. Interestingly, the directed FC from the left HIP to right MTG was significantly decreased in MCI patients only, and the directed FC from left MTG to right HIP was significantly decreased in AD patients only. Many studies have shown that the MTG plays an important role in language function (Mohammed et al., 2015; Khazaee et al., 2017) and that the gray matter volume of the MTG in MCI patients was significant different than that in controls. Consistent with these findings, Yan et al. (2013) found decreased effective connectivity among MTG and the HIP in patients with MCI, compared with controls, and this impaired connectivity was associated with patients’ cognitive performance in an auditory verbal learning task. Therefore, there may exist a compensation mechanism and the influence inconsistency of left and right MTG for the language function, in the process of developing from MCI to AD. In addition, comparing with NCs, the directed FCs from the left HIP to right PHG were significantly decreased in MCI patients, and the directed FC from the right HIP to left STG was significantly decreased in AD patients. The PHG plays an essential role in memory generation as a bridge between the HIP and cortex (Bar et al., 2008), and the STG has been reported to be a key region in emotional perception caused by facial stimulation and processing of auditory language and has been implicated as a critical structure in social cognition (Bigler et al., 2007). These changes might be related to the clinical manifestation of cognitive impairment in AD patients with dementia.

### Abnormal Directed Connectivity Between the HIP and the Executive Control Network

The directed FCs between the HIP and selected brain regions in the executive control network (ECN), such as SFG, MFG, and IFG, were abnormal. Consistent with our finding, a body of research has demonstrated that functional brain activity within portions of the ECN was abnormal in patients with MCI and AD (Firbank et al., 2016; Zhao et al., 2018). Generally, the ECN is considered responsible for the extrinsic awareness that regulates the executive functions that control and mediate cognitive processes, including working memory, reasoning, flexibility, problem solving, and planning (Zhao et al., 2018; Brown et al., 2019).

Specifically, the directed FCs from the left HIP to right SFG and from left MFG to the right HIP were significantly decreased in MCI and AD patients. In fact, the SFG (Khazaee et al., 2015) and the MFG (Levine et al., 2015; Liu et al., 2015) are important components of the dorsolateral prefrontal cortex that play an essential role in visual working memory (Oliveri et al., 2001). Furthermore, Cai et al. (2017) found that the dorsolateral prefrontal cortex and medial prefrontal cortex were the core nodes in the ECN and that they showed different connection patterns in patients with MCI and AD. Evidently, these changes in connectivity might explain why AD patients increasingly suffer from disruptions in visuospatial skills and memory impairment. Notably, the directed FC from the left HIP to right IFGtriang was significantly increased in MCI patients only, whereas the significant increase of this directed FC was observed in the opposite direction in AD patients. This may indicate that IFGtriang has a regulatory effect on cognitive control and memory retrieval in patients with AD.

### Abnormal Directed Connectivity Between the HIP and the Salience Network

As the functional module that plays a vital role in communication, social behavior, and self-awareness (Uddin, 2017), the salience network (SN) and its interactions with the HIP showed significant abnormalities. The SN is a large-scale paralimbic–limbic network anchored in the anterior insula and dorsal anterior cingulate cortex, with prominent subcortical nodes in the affect and reward processing systems (Menon, 2015). He et al. (2014) and Chand et al. (2017) found that the structure and function of the SN was disrupted in patients with MCI and AD. These results also indicated that the effective connections between the SN and other brain regions in MCI and AD patients may be different than those in NCs. Hence, the disruption in the SN found in this study might be associated with cognitive decline in patients with MCI and AD.

Notably, the directed FC from the left HIP to left ACG was significantly enhanced in MCI patients but obviously weakened in AD patients. Nevertheless, the directed FC from left ACG to the HIP was visibly increased in AD patients. These findings indicate that a compensatory mechanism is present during the development of AD pathology. In fact, according to research by Apps M A, the ACG plays an important role in monitoring the outcomes of decisions in uncertain or volatile environments and might be an advanced control structure responsible for behavior planning and the execution of executive network functions (Apps et al., 2009).

More interestingly, the directed FC from the HIP to right INS in AD patients showed a consistent weakening trend compared with that in NCs. Nevertheless, directed FC from right INS to the right HIP was significantly increased in patients with AD compared to those with MCI. These findings may be similar to the compensatory mechanism described above and may be important because the INS is usually considered to play an important role in emotional regulation and the control of body balance. Moreover, Kurth et al. (2010) suggested that the INS is related to social emotion, sensorimotor function, sense of smell and taste, and cognitive functions. Hence, the unusual interaction between the HIP and INS might be one reason why patients with AD showed changes in personality and behavior, cognitive decline, and executive dysfunction.

### Abnormal Directed Connectivity Between the HIP and Other Brain Regions

The current study found the directed FC from right SMA to the left HIP was increased in MCI patients. Consistent with these results, a previous study had reported the gradual loss of the sensorimotor cortex with the progression of AD (Frisoni et al., 2009), and functional alterations in the SMA in AD patients have been demonstrated in rs-fMRI studies (Brier et al., 2012). Moreover, the directed FC from the right HIP to left PCL decreased in MCI patients, and the directed FC from right PCL to the right HIP decreased in AD patients. These changes might explain symptoms such as apraxia and executive function in late AD.

Notably, our study showed that the directed FCs between the HIP and other brain regions in patients with MCI had been disrupted to a certain degree but that there were only abnormal connections with few brain regions. Nevertheless, in AD patients, the mass of affected voxels was larger, the number of widely distributed brain regions was larger, and the higher levels of statistical significance than MCI patients. These results might mean that during the transition from MCI to AD, a large number of brain regions become impaired, the directed FCs between the HIP and a large number of brain regions become disrupted, and the direction and intensity of information flow becomes altered, which might lead to clinical manifestations such as memory deficits, cognitive decline, and executive dysfunction in AD patients.

Additionally, one of the interesting findings was that the abnormal brain regions associated with the left HIP in MCI patients were primarily from the brain regions which receive information from the left HIP, whereas the abnormal brain regions associated with the left HIP in the AD patients were primarily from the brain regions which transmit information to the left HIP. This difference might be an imaging marker for the discrimination between MCI and AD and requires further study. Moreover, the current study showed that almost all brain regions with abnormal connectivity presented with unilateral abnormalities, with some exceptions. The abnormal FC was asymmetric in the two hemispheres, which was consistent with previous studies (Kim et al., 2012; Wachinger et al., 2018).

### Correlations Between Abnormal Directed Connectivity and Cognitive and Clinical Measurements

We performed correlation analyses between the GCA coefficients for the abnormal directional FCs and cognitive and clinical measurements (MMSE, CDR, and FAQ). These three cognitive and clinical measurements provide a quantitative assessment of cognitive function and are widely used (Ciesielska et al., 2016; Kaur et al., 2016; Kim et al., 2017). The higher the MMSE scores indicate the higher the cognitive ability; the lower functional performance is associated with higher FAQ scores, and the higher CDR scores indicate the presence of dementia. Overall, most of these abnormal directional FCs were significantly correlated with MMSE, CDR, and FAQ. Specifically, the directed FC from the left HIP to left ITG and MMSE were significantly negatively correlated, which indicated that the lower the MMSE, the stronger the FC. This is also consistent with our finding that this abnormal directed FC might be associated with cognitive decline in MCI and AD patients. Moreover, the directed FCs from the left HIP to left MFG and from the right HIP to left STG were significantly negatively correlated with CDR and FAQ in the three groups. This is not only consistent with the results of our discussion above, but also indicates that the patient’s visual working memory function is gradually decreasing. More consistently, the directed FC from the left HIP to left ACG was significantly positively correlated with CDR and FAQ in MCI patients, and was significantly negatively correlated with CDR and FAQ in AD patients. This indicates that the compensatory mechanism occurs in the brain during disease progression.

### Limitation

A limitation of this study is that the number of subjects used to assess the test–retest reliability of the abnormal directed FC is small, which may lead to potential instability of ICC values. In this study, we performed cross-group merging on the retest subjects and performed ICC analysis. We found fair average reliability of the abnormal directional FCs. Nevertheless, some abnormal directional FCs still have low reliability. This may be due to the small number of objects and the large between-subject mean square. The results in need for further validation with a larger dataset.

## Conclusion

In conclusion, this study reveals significant differences in directed FC between the hippocampus and other brain regions among NC, MCI, and AD subjects, and these differences were significantly correlated with cognitive and clinical measurements. These results might indicate patterns of alterations in the transmission and reception of information with the evolution of the disease. The analysis of abnormal directed connectivity might provide an additional target for the prevention and treatment of AD. Moreover, selected alterations could be reliable and used as discriminant features to classify NC, MCI, and AD groups. These possibilities require further study.

## Alzheimer’s Disease Neuroimaging Initiative

The data used in the preparation of this article were obtained from the Alzheimer’s Disease Neuroimaging Initiative (ADNI) database (adni.loni.usc.edu). As such, the investigators within the ADNI contributed to the design and implementation of the ADNI and/or provided data but did not participate in the analysis or writing of this report. A complete listing of the ADNI investigators can be found at https://adni.loni.usc.edu/wp-content/uploads/how_to_apply/ADNI_Acknowledgement_List.pdf.

## Data Availability Statement

All datasets generated for this study are included in the article/Supplementary Material.

## Ethics Statement

The studies involving human participants were reviewed and approved by The Alzheimer’s Disease Neuroimaging Initiative (ADNI) database. The patients/participants provided their written informed consent to participate in this study.

## Author Contributions

JXu performed the experiment and completed the manuscript. HG, HC, ZC, XW, and YG provided suggestions for this study. BW and JXi provided guidance throughout the study.

## Conflict of Interest

The authors declare that the research was conducted in the absence of any commercial or financial relationships that could be construed as a potential conflict of interest.

## Footnotes

1 http://adni.loni.ucla.edu

2 http://www.fil.ion.ucl.ac.uk/spm

3 http://restfmri.net/forum/REST_V1.8
