# Supplementary material for: Altered Directed Functional Connectivity of the Hippocampus in Mild Cognitive Impairment and Alzheimer’s Disease: A Resting-State fMRI Study
Source: Front Aging Neurosci. 2019 Dec 3;11:326. doi: 10.3389/fnagi.2019.00326 (PMC6905409; doi:10.3389/fnagi.2019.00326)

Altered directed functional connectivity of the hippocampus in mild cognitive impairment and Alzheimer’s disease: A resting-state fMRI study

Jiayue Xue^1†^, Hao Guo^1†^, Yuan Gao^1^, Xin Wang^1^, Huifang Cui^1^, Zeci Chen^1^, Bin Wang^1*^, Jie Xiang^1*^

^1^College of Information and Computer, Taiyuan University of Technology, Taiyuan, China

**†These authors are co-first authors**

*** Correspondence:**Jie Xiang, Email: xiangjie@tyut.edu.cn

Bin Wang, Email: wangbin01@tyut.edu.cn

## Supplementary Table

**Table S1** The average GCA coefficients for the abnormal directional connections between the left hippocampus and the whole brain within each group (NC, MCI and AD).

| **Brain Region** | **Abbreviation** | **NC** | | **MCI** | | **AD** | |  |
| --- | --- | --- | --- | --- | --- | --- | --- | --- |
| **Left HIP to the whole brain** |  |  | |  | |  | |  |
| Right Inferior temporal gyrus | ITG.R | 0.0108 | | -0.0809 | | -0.0247 | |  |
| Left Inferior temporal gyrus | ITG.L | -0.2539 | | 0.1440 | | 0.1595 | |  |
| Right Middle temporal gyrus | MTG.R | 0.0445 | | -0.0208 | | -0.0038 | |  |
| Right Parahippocampal gyrus | PHG.R | 0.0157 | | -0.1879 | | -0.1110 | |  |
| Left Inferior frontal gyrus, triangular portion | IFGtriang.L | 0.0404 | | -0.0909 | | 0.0047 | |  |
| Left Anterior cingulate and paracingulate gyri | ACG.L | -0.1798 | | 0.0782 | | -0.2430 | |  |
| Right Inferior frontal gyrus, triangular portion | IFGtriang.R | 0.0425 | | 0.1095 | | 0.0653 | |  |
| Left Middle frontal gyrus | MFG.L | 0.2382 | | 0.1028 | | 0.1704 | |  |
| Right Superior frontal gyrus | SFG.R | -0.1077 | | -0.2056 | | -0.2324 | |  |
| Right Temporal pole: superior temporal gyrus | TPOsup.R | 0.2956 | | 0.1739 | | -0.1760 | |  |
| Right Caudate nucleus | CAU.R | -0.1947 | | -0.2582 | | -0.3499 | |  |
| Right Posterior cingulate gyrus | PCG.R | 0.1465 | | 0.0560 | | -0.0266 | |  |
| Left Precuneus | PCUN.L | -0.0287 | | 0.0477 | | 0.1686 | |  |
| Left Temporal pole: superior temporal gyrus | TPOsup.L | 0.1352 | | 0.2067 | | -0.3341 | |  |
| Right Insula | INS.R | 0.0568 | | 0.1789 | | -0.0964 | |  |
| Right Cerebellum_6 | Cereb.R | 0.0561 | | 0.0115 | | 0.0994 | |  |
| Left Middle occipital gyrus | MOG.L | 0.0041 | | -0.0427 | | 0.0771 | |  |
| Right Thalamus | THA.R | 0.2582 | | 0.1488 | | 0.3954 | |  |
| Right Rolandic operculum | ROL.R | 0.1224 | | 0.1681 | | 0.0285 | |  |
| Left Superior frontal gyrus | SFG.L | 0.1028 | | 0.1427 | | 0.0178 | |  |
| **Whole brain to the left HIP** |  |  | |  | |  | |  |
| Right Inferior temporal gyrus | ITG.R | 0.1298 | | 0.2016 | | 0.1806 | |  |
| Right Inferior frontal gyrus, orbital portion | ORBinf.R | | -0.0591 | | 0.1004 | | 0.0269 | |
| Left Superior frontal gyrus, medial | SFGmed.L | | 0.2111 | | 0.0265 | | 0.0772 | |
| Right Supplementary motor area | SMA.R | | -0.2984 | | 0.1031 | | -0.0006 | |
| Left Cerebellum_9 | Cereb.L | | 0.0663 | | 0.1042 | | -0.2029 | |
| Right Cerebellum_6 | Cereb.R | | 0.1325 | | 0.0937 | | 0.0020 | |
| Left Cerebellum_6 | Cereb.L | | 0.0536 | | 0.1529 | | -0.3685 | |
| Left Inferior temporal gyrus | ITG.L | | 0.1298 | | 0.0600 | | 0.0005 | |
| Right Inferior frontal gyrus, triangular portion | IFGtriang.R | | -0.0591 | | 0.0242 | | 0.1922 | |
| Right Caudate nucleus | CAU.R | | -0.1650 | | -0.1531 | | -0.0293 | |
| Right Superior frontal gyrus | SFG.R | | -0.0477 | | 0.0353 | | 0.1953 | |
| Left Cerebellum_Crus2 | Cerebe_Crus2.L | | 0.0247 | | 0.1155 | | -0.1361 | |
| Left Cerebellum_Crus1 | Cerebe_Crus1.L | | 0.0553 | | 0.0731 | | -0.0962 | |
| Left Anterior cingulate and paracingulate gyri | ACG.L | | 0.2915 | | -0.0216 | | 0.3589 | |
| Left Superior frontal gyrus | SFG.L | | 0.0495 | | 0.0184 | | 0.1050 | |
| Left Inferior frontal gyrus, triangular portion | IFGtriang.L | | 0.0861 | | 0.0606 | | 0.1279 | |

## Table S2 The average GCA coefficients for the abnormal directional connections between the right hippocampus and the whole brain within each group (NC, MCI and AD).

| **Brain Region** | **Abbreviation** | **NC** | **MCI** | **AD** |
| --- | --- | --- | --- | --- |
| **Right HIP to the whole brain** |  |  |  |  |
| Right Lenticular nucleus, pallidum | PAL.R | 0.1126 | -0.0757 | 0.0164 |
| Left Calcarine fissure and surrounding cortex | CAL.L | 0.1069 | 0.1567 | 0.1188 |
| Right Middle frontal gyrus | MFG.R | -0.1603 | 0.1189 | 0.1292 |
| Left Paracentral lobule | PCL.L | 0.0516 | -0.1984 | -0.0319 |
| Right Insula | INS.R | 0.1880 | 0.0923 | -0.2185 |
| Left Inferior frontal gyrus, triangular portion | IFGtriang.L | -0.1079 | -0.1419 | 0.0194 |
| Left Superior temporal gyrus | STG.L | 0.3001 | 0.2393 | 0.0132 |
| Right Median cingulate and paracingulate gyri | DCG.R | 0.0116 | -0.0029 | 0.0635 |
| Left Precentral gyrus | PreCG.L | 0.0065 | 0.0297 | 0.0449 |
| Left Inferior parietal, but supramarginal and angular gyri | IPL.L | 0.0930 | 0.1794 | 0.2392 |
| Right Precuneus | PCUN.R | 0.0608 | -0.0339 | 0.1799 |
| Right Heschl gyrus | HES.R | 0.2498 | 0.3804 | -0.4150 |
| Right Fusiform gyrus | FFG.R | -0.0203 | -0.0650 | 0.0727 |
| Left Insula | INS.L | -0.0496 | -0.0009 | -0.1196 |
| Right Caudate nucleus | CAU.R | 0.1132 | 0.2173 | -0.2093 |
| Left Caudate nucleus | CAU.L | 0.1151 | 0.3540 | 0.0090 |
| **Whole brain to the right HIP** |  |  |  |  |
| Right Cerebellum_Crus2 | Cerebe_Crus2.R | 0.0339 | -0.0114 | -0.0048 |
| Right Heschl gyrus | HES.R | -0.4621 | -0.2354 | 0.2423 |
| Left Middle temporal gyrus | MTG.L | 0.1040 | 0.0672 | 0.0070 |
| Right Calcarine fissure and surrounding cortex | CAL.R | -0.2491 | -0.1064 | 0.0210 |
| Left Anterior cingulate and paracingulate gyri | ACG.L | 0.1825 | 0.1515 | 0.2653 |
| Left Middle frontal gyrus | MFG.L | 0.1683 | 0.1026 | -0.0649 |
| Right Superior occipital gyrus | SOG.R | -0.0208 | -0.1416 | -0.1894 |
| Right Insula | INS.R | -0.0559 | -0.2798 | 0.1857 |
| Right Inferior frontal gyrus, opercular portion | IFGoperc.R | -0.0178 | -0.1031 | 0.0909 |
| Right Precuneus | PCUN.R | -0.1058 | 0.0045 | -0.1766 |
| Right Paracentral lobule | PCL.R | -0.0234 | 0.0821 | -0.0619 |

**Table S3** Correlations between the GCA coefficients for abnormal directional connections between the left hippocampus and the whole brain and cognitive and clinical measurement scores (MMSE, FAQ and CDR) in the three groups (*p* < 0.05, corrected). *r* is the Spearman correlation coefficient, and *p* indicates the level of statistical significance.

| **Brain Region** | | **Abbreviation** | **MMSE(r,p)** | **CDR(r,p)** | | **FAQ(r,p)** |
| --- | --- | --- | --- | --- | --- | --- |
| **Left HIP to the whole brain** | |  |  |  | |  |
| **NC vs. MCI** | |  |  |  | |  |
| Right Inferior temporal gyrus | | ITG.R | **(0.219, 0.038*)** | (-0.148, 0.164) | | (-0.043, 0.685) |
| Left Inferior temporal gyrus | | ITG.L | **(-0.214,0.045*)** | (0.204,0.054) | | (0.023, 0.829) |
| Right Middle temporal gyrus | | MTG.R | (0.178, 0.093) | (-0.148,0.163) | | **(-0.265, 0.012*)** |
| Right Parahippocampal gyrus | | PHG.R | (0.133, 0.210) | (-0.132,0.217) | | (-0.080, 0.452) |
| Left Inferior frontal gyrus, triangular portion | | IFGtriang.L | (0.101, 0.344) | (-0.036,0.736) | | (-0.116, 0.276) |
| Left Anterior cingulate and paracingulate gyri | | ACG.L | (-0.032, 0.762) | **(0.286,0.006**)** | | **(0.301, 0.004**)** |
| Right Inferior frontal gyrus, triangular portion | | IFGtriang.R | (-0.070, 0.513) | (0.171,0.107) | | (0.071, 0.508) |
| Left Middle frontal gyrus | | MFG.L | (0.145, 0.276) | **(-0.243,0.021*)** | | **(-0.239, 0.027*)** |
| Right Superior frontal gyrus | | SFG.R | (0.025, 0.813) | (-0.113,0.288) | | (-0.028, 0.795) |
| **NC vs. AD** | |  |  |  |  | |
| Right Temporal pole: superior temporal gyrus | | TPOsup.R | **(0.292,0.029*)** | **(-0.264,0.049*)** | | (-0.231,0.087) |
| Left Inferior temporal gyrus | | ITG.L | **(-0.330,0.013*)** | (0.262,0.051) | | (0.220,0.103) |
| Right Caudate nucleus | | CAU.R | (0.157,0.249) | (-0.158,0.246) | | (-0.139,0.306) |
| Right Posterior cingulate gyrus | | PCG.R | (0.008,0.951) | (-0.056,0.683) | | (-0.130,0.338) |
| Right Superior frontal gyrus | | SFG.R | (0.064,0.640) | (-0.030,0.828) | | (-0.024,0.859) |
| Left Precuneus | | PCUN.L | **(-0.300,0.025*)** | (0.204,0.132) | | (0.088,0.517) |
| **MCI vs. AD** | |  |  |  |  | |
| Left Temporal pole: superior temporal gyrus | | TPOsup.L | (0.096,0.370) | (-0.113,0.288) | | (-0.127,0.233) |
| Right Insula | | INS.R | **(0.224,0.031*)** | (-0.174,0.100) | | (-0.084,0.432) |
| Right Cerebellum_6 | | Cereb.R | (-0.056,0.599) | (0.037,0.730) | | (0.103,0.332) |
| Left Middle occipital gyrus | | MOG.L | (-0.147,0.165) | **(0.298,0.025*)** | | **(0.238,0.026*)** |
| Right Thalamus | | THA.R | **(-0.291,0.028*)** | (0.115,0.282) | | (0.139,0.193) |
| Right Rolandic operculum | | ROL.R | (0.049,0.649) | (-0.096,0.367) | | (-0.039,0.712) |
| Left Anterior cingulate and paracingulate gyri | | ACG.L | **(0.317,0.002*)** | **(-0.223,0.034*)** | | (-0.199,0.060) |
| Left Superior frontal gyrus | | SFG.L | (0.060,0.575) | (-0.052,0.627) | | (-0.041,0.704) |
| **Whole brain to the left HIP** | |  |  |  | |  |
| **NC vs. MCI** | |  |  |  | |  |
| Right Inferior temporal gyrus | | ITG.R | **(-0.215,0.042*)** | (0.143,0.179) | | (0.127,0.232) |
| Right Inferior frontal gyrus, orbital portion | ORBinf.R | | (-0.049,0.643) | (0.099,0.352) | | (0.044,0.678) |
| Left Superior frontal gyrus, medial | SFGmed.L | | **(0.254,0.018*)** | (-0.071,0.509) | | (-0.087,0.414) |
| Right Supplementary motor area | SMA.R | | (-0.138,0.195) | (0.181,0.088) | | (0.120,0.261) |
| **NC vs. AD** |  | |  |  | |  |
| Left Cerebellum_9 | Cereb.L | | (0.174,0.200) | (-0.261,0.052) | | (-0.247,0.066) |
| Left Cerebellum_Crus2 | Cereb.L | | **(0.266,0.048*)** | **(-0.310,0.020*)** | | **(-0.393,0.003**)** |
| Right Cerebellum_6 | Cereb.R | | (0.257,0.056) | **(-0.295,0.027*)** | | **(-0.381,0.004**)** |
| Left Cerebellum_6 | Cereb.L | | (0.225,0.095) | (-0.255,0.058) | | (-0.228,0.090) |
| Left Inferior temporal gyrus | ITG.L | | (0.191,0.160) | (-0.244,0.070) | | (-0.126,0.355) |
| Right Inferior frontal gyrus, triangular portion | IFGtriang.R | | **(-0.322,0.015*)** | **(0.345,0.009**)** | | **(0.319,0.016*)** |
| Right Caudate nucleus | CAU.R | | (-0.030,0.824) | (0.101,0.461) | | (0.084,0.540) |
| Right Superior frontal gyrus | SFG.R | | **(-0.309,0.020*)** | **(0.337,0.011*)** | | **(0.330,0.013*)** |
| **MCI vs. AD** |  | |  |  | |  |
| Left Cerebellum_Crus2 | Cerebe_Crus2.L | | (0.205,0.053) | **(-0.256,0.015*)** | | **(-0.263,0.012*)** |
| Left Cerebellum_Crus1 | Cerebe_Crus1.L | | **(0.225,0.033*)** | **(-0.249,0.018*)** | | **(-0.293,0.005**)** |
| Left Anterior cingulate and paracingulate gyri | ACG.L | | (-0.198,0.061) | (0.113,0.289) | | (0.086,0.420) |
| Left Superior frontal gyrus | SFG.L | | (-0.202,0.057) | (0.123,0.249) | | (0.102,0.341) |
| Right Caudate nucleus | CAU.R | | (-0.011,0.917) | (0.075,0.482) | | (0.056,0.601) |
| Left Inferior frontal gyrus, triangular portion | IFGtriang.L | | **(-0.249,0.018*)** | (0.106,0.322) | | (0.136,0.200) |
| Right Inferior frontal gyrus, triangular portion | IFGtriang.R | | **(-0.243,0.021*)** | (0.110,0.302) | | (0.114,0.287) |

**Table S4** Correlations between the GCA coefficients for abnormal directional connections between the right hippocampus and the whole brain and cognitive and clinical measurement scores (MMSE, FAQ and CDR) in the three groups (*p* < 0.05, corrected). *r* is the Spearman correlation coefficient, and *p* indicates the level of statistical significance.

| **Brain Region** | **Abbreviation** | **MMSE(r,p)** | **CDR(r,p)** | **FAQ(r,p)** |
| --- | --- | --- | --- | --- |
| **Right HIP to the whole brain** |  |  |  |  |
| **NC vs. MCI** |  |  |  |  |
| Right Lenticular nucleus, pallidum | PAL.R | (0.082,0.444) | **(0.246,0.019*)** | (0.051,0.634) |
| Left Calcarine fissure and surrounding cortex | CAL.L | (-0.034,0.749) | (0.018,0.868) | (0.120,0.259) |
| Right Middle frontal gyrus | MFG.R | (-0.119,0.263) | **(0.284,0.035*)** | **(0.215,0.044*)** |
| Left Paracentral lobule | PCL.L | (0.019,0.862) | (-0.129,0.225) | (-0.048,0.653) |
| **NC vs. AD** |  |  |  |  |
| Left Vermis_10 | Vermis_10. L | (0.049,0.722) | (-0.063,0.644) | (-0.048,0.727) |
| Right Insula | INS.R | **(0301,0.024*)** | **(-0.314,0.018*)** | (-0.251,0.062) |
| Left Inferior frontal gyrus, triangular portion | IFGtriang.L | (-0.065,0.636) | (0.190,0.160) | (0.156,0.252) |
| Left Superior temporal gyrus | STG.L | (0.056,0.682) | **(-0.300,0.025)** | **(-0.337,0.011*)** |
| Right Middle frontal gyrus | MFG.R | **(-0.286,0.033*)** | **(0.366,0.006**)** | **(0.310,0.020*)** |
| Right Median cingulate and paracingulate gyri | DCG.R | (-0.104,0.444) | (0.075,0.581) | (0.032,0.816) |
| Left Precentral gyrus | PreCG.L | (-0.065,0.635) | (0.032,0.813) | (0.132,0.331) |
| Left Inferior parietal, but supramarginal and angular gyri | IPL.L | **(-0.264,0.049*)** | (0.101,0.461) | (0.033,0.811) |
| Right Precuneus | PCUN.R | **(-0.331,0.013*)** | **(0.271,0.043*)** | (0.242,0.072) |
| **MCI vs. AD** |  |  |  |  |
| Right Heschl gyrus | HES.R | (0.105,0.325) | **(-0.244,0.020*)** | (-0.173,0.104) |
| Right Fusiform gyrus | FFG.R | (-0.140,0.188) | (0.085,0.427) | (0.081,0.449) |
| Left Insula | INS.L | **(0.217,0.045*)** | (-0.152,0.152) | (-0.045,0.670) |
| Left Inferior frontal gyrus, triangular portion | IFGtriang.L | (-0.009,0.932) | (0.176,0.097) | (0.106,0.319) |
| Right Caudate nucleus | CAU.R | (0.102,0.338) | (-0.087,0.415) | (-0.059,0.581) |
| Left Caudate nucleus | CAU.L | **(0.215,0.044*)** | (-0.108,0.310) | (-0.065,0.545) |
| Left Superior temporal gyrus | STG.L | (0.029,0.784) | **(-0.226,0.032*)** | **(-0.212,0.044*)** |
| Right Median cingulate and paracingulate gyri | DCG.R | (-0.098,0.361) | (0.062,0.563) | (0.031,0.768) |
| Right Precuneus | PCUN.R | **(-0.274,0.009**)** | **(0.247,0.019*)** | (0.188,0.076) |
| **Whole brain to the right HIP** |  |  |  |  |
| **NC vs. MCI** |  |  |  |  |
| Right Cerebellum_Crus2 | Cerebe_Crus2.R | (-0.070,0.510) | (0.038,0.722) | (0.150,0.158) |
| **NC vs. AD** |  |  |  |  |
| Right Cerebellum_Crus2 | Cerebe_Crus2.R | (0.173,0.203) | (-0.262,0.051) | **(-0.339,0.011*)** |
| Right Heschl gyrus | HES.R | (-0.114,0.401) | **(0.352,0.008**)** | **(0.348,0.009**)** |
| Left Middle temporal gyrus | MTG.L | (0.047,0.730) | (-0.020,0.886) | (-0.049,0.719) |
| Right Calcarine fissure and surrounding cortex | CAL.R | (-0.204,0.132) | **(0.278,0.038*)** | (0.184,0.075) |
| Left Anterior cingulate and paracingulate gyri | ACG.L | (-0.034,0.804) | (0.056,0.682) | (0.007,0.957) |
| Left Middle frontal gyrus | MFG.L | **(0.403,0.002**)** | **(-0.384,0.003**)** | **(-0.352,0.008**)** |
| Right Superior occipital gyrus | SOG.R | (0.060,0.661) | (-0.000,0.999) | (-0.090,0.508) |
| **MCI vs. AD** |  |  |  |  |
| Right Insula | INS.R | (-0.036,0.739) | (0.163,0.125) | (0.079,0.459) |
| Left Anterior cingulate and paracingulate gyri | ACG.L | (-0.118,0.267) | (0.064,0.550) | (0.002,0.986) |
| Left Superior temporal gyrus | STG.L | (-0.009,0.931) | **(0.284,0.035*)** | **(0.258,0.015*)** |
| Right Inferior frontal gyrus, opercular portion | IFGoperc.R | (-0.053,0.622) | (0.026,0.804) | (0.035,0.741) |
| Right Precuneus | PCUN.R | (0.145,0.173) | **(-0.215,0.042*)** | **(-0.251,0.017*)** |
| Left Middle frontal gyrus | MFG.L | (0.156,0.141) | **(-0.222,0.037*)** | (-0.199,0.061) |
| Right Paracentral lobule | PCL.R | (0.106,0.320) | (-0.033,0.757) | (-0.038,0.725) |

## Supplementary Figure

**Figure S1** Test-retest reliability of directed functional connectivity from the left hippocampus to the whole brain and from the whole brain to the left hippocampus between each pair of groups. Figure S1A is the ICC results for the MCI and NC groups, Figure S1B is the ICC results for the AD and NC groups, and Figure S1C is the ICC results for the AD and MCI groups. ICC values from 0 to 1 are mapped in dark blue to dark red. The solid line indicates the directed functional connectivity from the left hippocampus to other brain regions, and the dashed line indicates the directed functional connectivity from other brain regions to the left hippocampus.


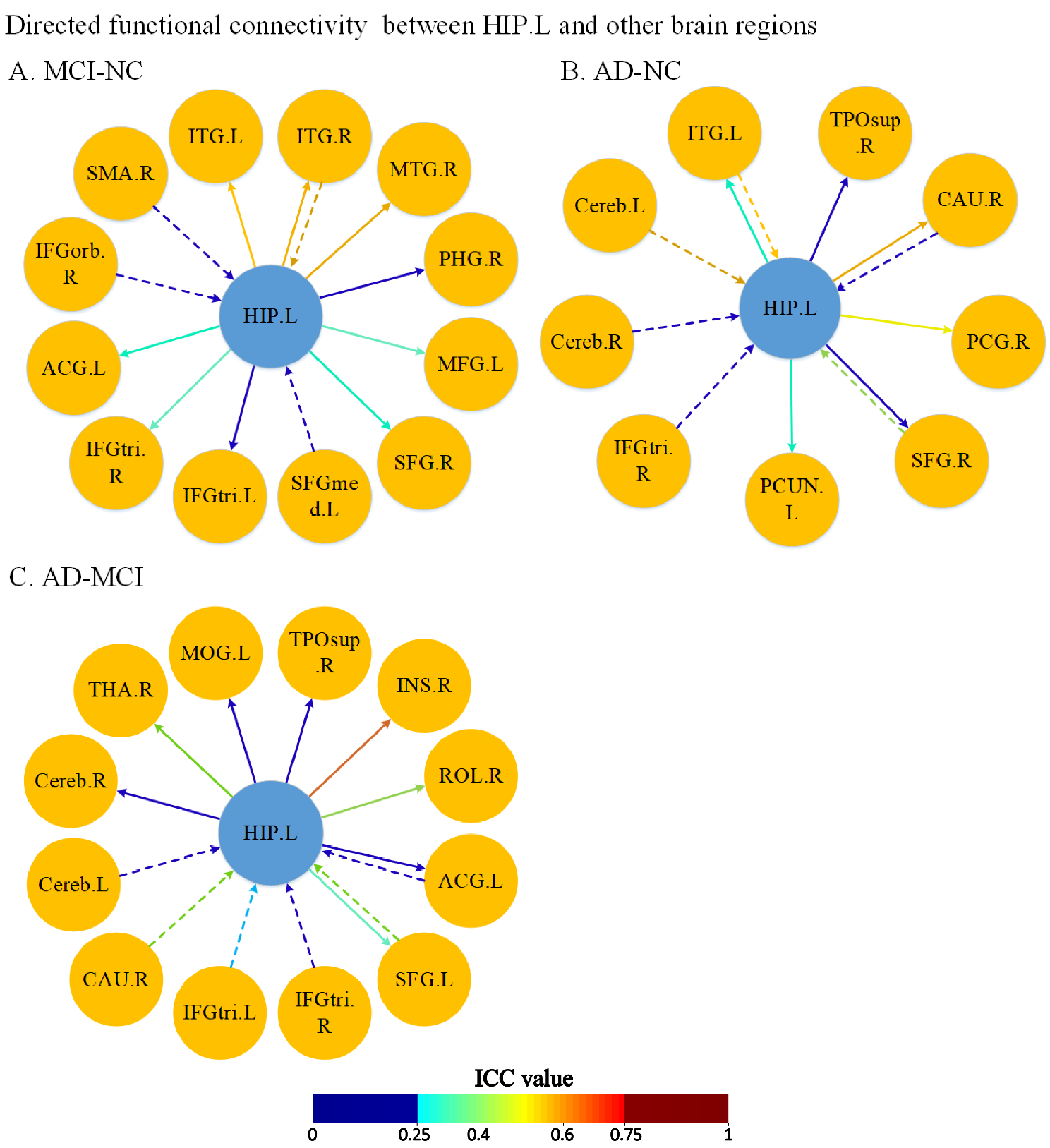


**Figure S2** Test-retest reliability of directed functional connectivity from the right hippocampus to the whole brain and from the whole brain to the right hippocampus between each pair of groups. Figure S2A is the ICC results for the MCI and NC groups, Figure S2B is the ICC results for the AD and NC groups, and Figure S2C is the ICC results for the AD and MCI groups. ICC values from 0 to 1 are mapped in dark blue to dark red. The solid line indicates the directed functional connectivity from the right hippocampus to other brain regions, and the dashed line indicates the directed functional connectivity from other brain regions to the right hippocampus.


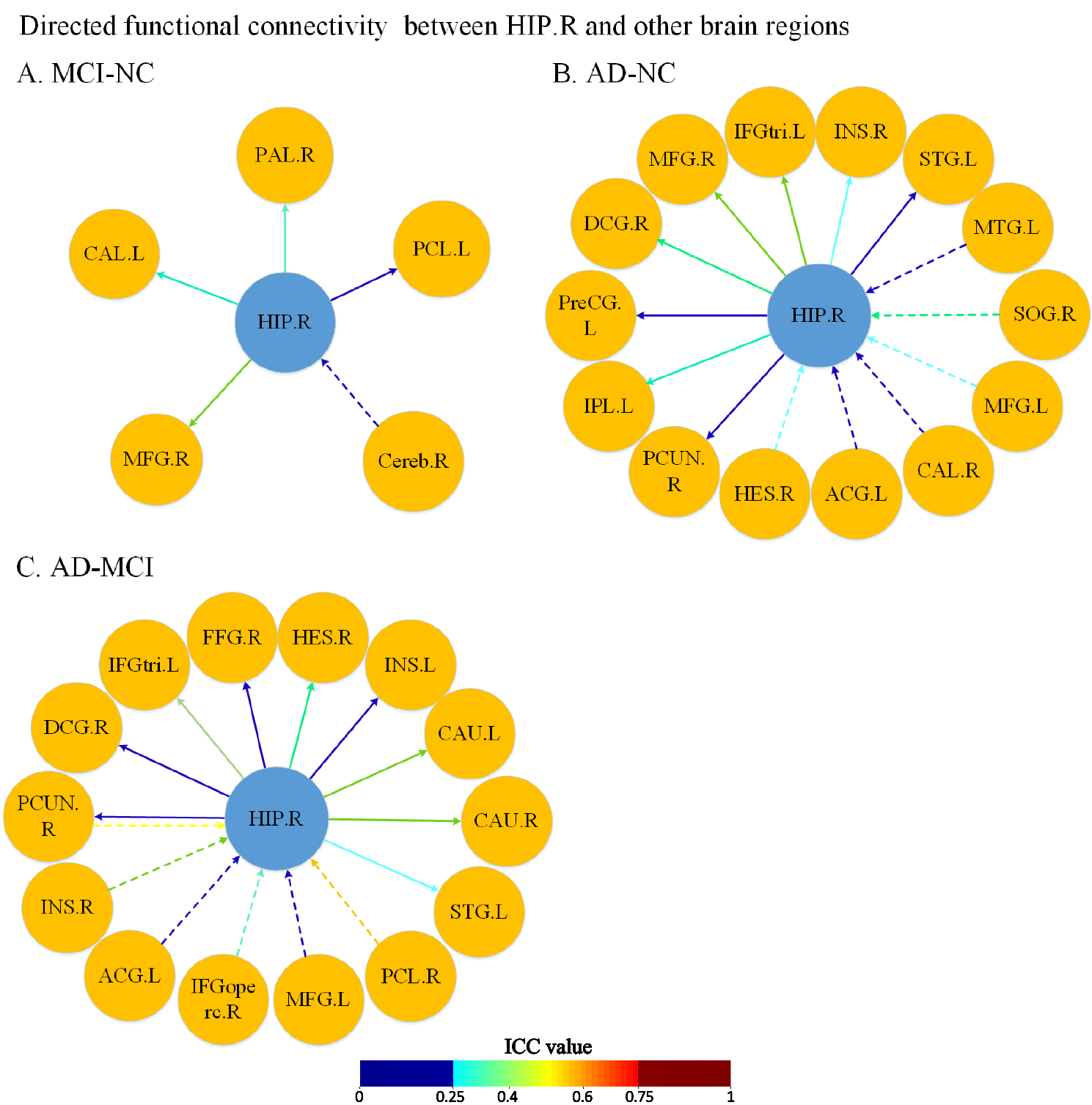

Supplement: Supplementary file 1 [file Data_Sheet_1.docx]
